# Extracellular vesicles derived from lipoaspirate fluid promote fat graft survival

**DOI:** 10.1080/21623945.2021.1932355

**Published:** 2021-06-01

**Authors:** Fangfei Nie, Pengbing Ding, Chen Zhang, Zhenmin Zhao, Hongsen Bi

**Affiliations:** From the Department of Plastic Surgery, Peking University Third Hospital; Beijing, P.R. China

**Keywords:** Extracellular vesicles, adipose tissue, tumescent liposuction, fat graft, angiogenesis, adipogenesis

## Abstract

Extracellular vesicles (EVs) are specific subcellular vesicles released by cells under various environmental conditions. Tumescent liposuction is a commonly used procedure in plastic surgery practice. In the present study, we aimed to extract EVs derived from lipoaspirate fluid (LF-EVs) and characterize them using transmission electron microscopy, nanoparticle tracking analysis, and western blotting. The global profiles of proteins and microRNAs from LF-EVs were identified, strongly suggesting a potential regulatory function of LF-EVs. In addition, we investigated the effects and mechanisms of LF-EVs on fat graft survival. Cell functional tests showed that LF-EVs promoted the proliferation, migration, and tube structure formation of human umbilical vein endothelial cells. LF-EVs also promoted the adipogenic differentiation of adipose tissue-derived stem cells. The results of animal experiments showed that the average weights of fat grafts in the LF-EVs-treated group were significantly higher than those in the control group. Histologically, there was less fibrosis, fewer cysts, and increased fat tissue survival in the LF-EVs group. Further investigations of angiogenic and adipogenic factors revealed that LF-EVs also promoted angiogenesis and exerted a pro-adipogenic effect *in vivo*. Our findings will help to elucidate the functions of LF-EVs and provide a reference dataset for future translational studies.

## Introduction

Extracellular vesicles (EVs) are cell-derived, membrane-bound phospholipid vesicles that are present in many biological fluids, including blood, urine, cerebrospinal fluid, and cell culture media. Although initially thought to be cellular debris, and thus underappreciated, EVs are now increasingly recognized as important vehicles of intercellular communication, exchanging DNA, RNA, proteins, and lipids between cells [1,2].

Studies have shown that EVs can be used in many fields of tissue repair and regenerative medicine, such as skin wounds, fat transplantation, scar inhibition, flap survival, and skin rejuvenation [3–8]. In the field of stem cell and regenerative medicine, the application of EVs reduces the risk of abnormal cell differentiation and tumorigenesis compared with the direct use of stem cells [9].

Traditionally, liposuction has been used mainly for body contouring and autologous fat transplantation. It has the advantages of rich sources, simple operation, and no rejection reaction. However, there is an unpredictable absorption rate, which often requires multiple injections to achieve satisfactory results. How to improve the survival of fat tissue after fat transplantation is one of the hot topics in plastic surgery. To improve the fat tissue survival rate, many methods have been proposed, including the use of adipose tissue-derived stem cells (ADSCs), exosomes derived from ADSCs, and ADSC exosomes pretreated with hypoxia [5,10].

EVs can shuttle specific proteins, lipids, mRNA, microRNAs (miRNAs), DNA, and other signal molecules to regulate biological behaviour. The content of EVs mainly reflects the cells they are derived from. The stromal vascular fraction (SVF) and mature adipocytes (MAs) are the two major components of adipose tissue. SVF cells isolated from adipose tissue contain a variety of cell types, including mesenchymal stem cells, endothelial progenitor cells, endothelial cells, pericytes, preadipocytes, B and T lymphocytes, monocytes, macrophages, and fibroblasts [11,12]. According to previous reports, there is no significant difference in the effect or mechanism between SVF cells and ADSCs in the wound healing process, and exosomes are comparable to ADSCs in terms of fat graft retention [13,14]. Cell-free components of adipose tissue extracted using various methods were effective in tissue repair or regeneration. Bellei et al. showed that the physiological secretome of adipose tissue, in the form of a liquid extracellular fraction of lipoaspirate, was an efficient agent for skin regeneration in an *in vitro* model [15]. Sarkanen et al. found that cell-free adipose tissue extract (ATE) could effectively induce adipogenesis [11,16]. Lu et al. and Zheng et al. confirmed the inductive effect of ATE on adipose tissue regeneration in animal experiments [17,18]. However, to date, there has been no study concerning the protein and miRNA profiles of EVs derived from lipoaspirate fluid (LF-EVs) and their effect on adipose tissue regeneration.

In the present study, we aimed to evaluate the effects of LF-EVs on fat transplantation, to investigate the underlying mechanisms, and to explore a new autogeneic component to assist fat transplantation.

## Materials and methods

### Sample collection and LF-EVs isolation

The present study was approved by, and performed in accordance with, the guidelines and study protocols of the Peking University Third Hospital Medical Science Research Ethics Committee (approval no. M2017378). The procedure of tumescent liposuction was as follows. A large amount of tumescent solution was injected into the subcutaneous fat tissue of the liposuction site rapidly. Subcutaneous adipose tissue and interstitial fluid were then sucked out using negative pressure suction and collected in a sterile container from the hips, outer thigh, waist, or abdomen of ten healthy women (aged 31.5 ± 7.2 years) with moderate body mass indexes (BMIs) (22.6 ± 2.9 kg/m^2^). Tumescence solution (per litre) contained 1000 mL of normal saline (0.9%, N.S.), 600 mg of lidocaine, 1 mg of epinephrine, and 10 ml of 5% sodium bicarbonate (6 mmol).

**Figure 1.**
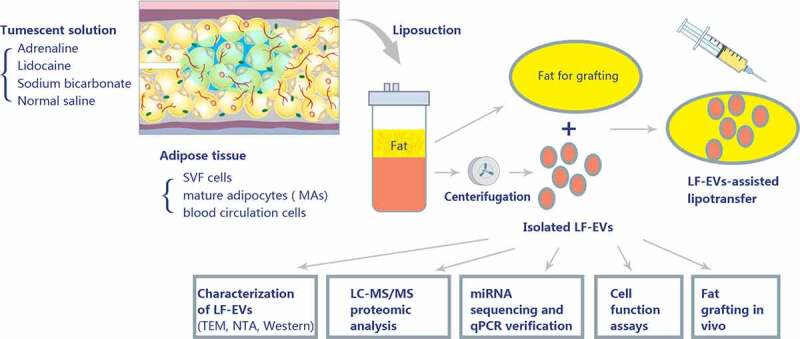
Schematic overview of the design of the study. A tumescent liposuction solution containing adrenaline, lidocaine, sodium bicarbonate, and normal saline was injected to subcutaneous adipose tissue. The main cell components of adipose tissue comprise stromal vascular fraction (SVF) cells, mature adipocytes (MAs), and blood circulation cells. Adipose tissue produces a large amount of extracellular vesicles (EVs) (referred to as EVs derived from lipoaspirate fluid (LF-EVs)) during the liposuction process. These LF-EVs were aspirated with the adipose tissue and tissue fluid into sterile containers. The LF-EVs were purified using sequential centrifugation and characterized by transmission electron microscopy (TEM), nanoparticle tracking analysis (NTA), and western blotting. The global profiles of proteins and miRNAs from LF-EVs were identified. The biological functions of LF-EVs *in vitro* and in LF-EVs-assisted lipotransfer *in vivo* were studied

The aseptic container with subcutaneous adipose tissue and interstitial fluid was transferred to the biosafety cabinet. The liquid components were set aside for LF-EVs extraction, and the adipose tissue was kept for nude mouse transplantation or ADSCs extraction. During the extraction of LF-EVs, all separation and extraction steps, except for centrifugation, were operated in the biosafety cabinet. The liquids were centrifuged at 300 × *g* for 5 minutes to remove the sediment and fat/oil layer. The middle fluid layer was collected and filtered through a 150-μm sterile filter. The fluid was initially centrifuged at 6,000 ×* g* for 6 min to remove the cell debris. The supernatant was then centrifuged at 12,000 × *g* for 12 min, followed by ultracentrifugation at 100,000 × *g* for 60 min to obtain the exosomes. The obtained pellets (LF-EVs) were stored at – 80°C. Before use, the LF-EVs were dissolved in 0.9% N.S. and filtered through a 0.22-μm filter (Corning Glass Works, Corning, NY, USA). A flow chart of the isolation and analyses is presented in Figure 1.

### Characterization of LF-EVs

The morphology and size of LF-EVs were observed using a JEM-1400 plus transmission electron microscope (JEOL, Tokyo, Japan). Nanoparticle tracking analysis (NTA) was performed using the ZetaView PMX 110 (Particle Metrix, Meerbusch, Germany) and its corresponding software (ZetaView 8.04.02 SP2), according to the manufacturer’s instructions. The protein concentration was determined using the bicinchoninic acid (BCA) protein assay kit (cat. No. CW0014S; CW biotech, Beijing, China). Western blotting analysis was performed using primary antibodies recognizing PDCD6IP (1:500; cat. No. 12,422-1-AP), CD9 (1:500; cat. No. 20,597-1-AP) and tumour susceptibility 101 (TSG101; 1:500; cat. No. 14,497-1-AP) from Proteintech Group, Inc. (Rosemont, IL, USA).

### Proteome analysis of LF-EVs

First, proteins were extracted from three individual LF-EVs samples using lysis buffer (2% SDS, 7 M Urea) containing protease inhibitors (cat. No. 78,429; Thermo Fisher Scientific, Waltham, MA, USA). After sonication and centrifugation, the protein content in the supernatant was analysed using the BCA assay. To remove non-protein substances, six volumes of 100% acetone was added and precipitated overnight at –20°C. After washing and centrifugation, the precipitates were dissolved and the protein concentration was quantified using the BCA assay again. Then, the sample was alkylated using 90 mM iodoacetamide in the dark at room temperature. The proteins were then subjected to trypsin digestion at 37°C overnight. Next day, the peptides were collected by centrifugation and dried using centrifugal concentration. Lastly, the peptides were desalted on a Ziptip C18 column, dried in a vacuum concentration apparatus, and stored at −20°C for liquid chromatography tandem mass spectrometry (LC-MS/MS) proteomic analysis.

The dried sample was dissolved in buffer (0.1% formic acid), and analysed by mass spectrometry. On-line Nano-reversed phase liquid chromatography (RPLC) was performed using the Easy-nLC 1200 system (Thermo Fisher Scientific). The sample was loaded onto a trap column (home-made C18, 100 μm × 2 cm, 5 μm particles) and further resolved on an analytical column (C18, 75 µm × 200 mm, 1.9 µm particles) at a flow rate of 200 nL/min. The peptides were then subjected to nano electrospray ionization source followed by tandem mass spectrometry in an Orbitrap Fusion Lumos instrument (Thermo Fisher Scientific). The mass spectrometer was operated in the data-dependent mode. Full-scan MS spectra were acquired with a resolution of 60,000, and the mass load ratio ranged from 370 to 1,600 m/z. In higher-energy collisional dissociation (HCD) fragmentation mode, the collision energy was set to 30%.

The proteomic data were analysed according to the MaxLFQ algorithm embedded in the MaxQuant software (MaxQuant 1.5.8.3, Max-Planck Institute for Biochemistry, Germany), and this quantitative value (Intensity) was calculated to obtain the normalized label-free quantitative value (LFQ).

## RNA isolation, miRNA sequencing, and qPCR verification of highly abundant miRNAs in LF-EVs

To investigate the miRNA content of LF-EVs, next-generation small RNA-sequencing (NGS RNA-seq) was carried out. Briefly, total RNA was extracted from LF-EVs of three individuals using a miRNeasy Serum/Plasma Kit (cat. No. 217,184; Qiagen, Hilden, Germany) following the manufacturer’s protocol. TruSeq Small RNA Sample Prep Kits (cat. No. RS-200-0024; Illumina, San Diego, CA, USA) were used to prepare the NGS RNA-seq library. To validate the library, we checked the size and purity of the sample on an Agilent Technologies 2100 Bioanalyzer (Agilent Technologies, Santa Clara, CA, USA). The qualified libraries were then sequenced on an Illumina Hiseq X Ten platform at OE biotech Co., Ltd. (Shanghai, China). The basic reads were converted into sequence data (also called raw data/reads) by base calling. First, we removed splice sequences from the raw reads, and then quality control of these sequences was carried out to remove the N-base sequences and the sequences with a low Q20 ratio. Reads shorter than 15 nt and longer than 41 nt were filtered out from the raw data, leaving the clean reads.

For primary analysis, the length distribution of the clean sequences in the reference genome was determined. Known miRNAs were identified by aligning against the miRBase v.21 database (http://www.mirbase.org/). Unannotated small RNAs were analysed using mirdeep2 (https://www.mdc-berlin.de/content/mirdeep2-documentation), to predict novel miRNAs.

Then, the expression of the top 20 highly expressed miRNAs screened through sequencing were verified in the LF-EVs from 10 individuals. Total RNA was extracted from LF-EVs of 10 individuals, as described above. After reverse transcription using the miScript Reverse Transcriptase Mix (cat. No. 218,161; Qiagen, Hilden, Germany), quantitative real-time PCR (qPCR) was performed using a LightCycler® 480 II Real-time PCR Instrument (Roche, Swiss) with the miScript SYBR Green PCR Kit (cat. No. 218,073; Qiagen, Hilden, Germany), and microRNA-specific primers (listed in Table 1). The 2^−ΔCT^ method was used to analyse the relative expression levels of microRNAs, normalized to the expression of U6.

**Table 1.** Top 20 miRNAs and their microRNA-specific primer sequences and relative expression quantification

microRNAs Name	Primer Sequence	Expression multiples relative to U6
U6	CAAGGATGACACGCAAATTCG	NA
hsa-let-7a-5p	GCGTGAGGTAGTAGGTTGTATA	55.14
hsa-let-7b-5p	TGAGGTAGTAGGTTGTGTGGTT	29.04
hsa-let-7 c-5p	TGAGGTAGTAGGTTGTATGGTT	15.07
hsa-miR-125b-5p	CCCTGAGACCCTAACTTGTGA	12.11
hsa-miR-486-5p	TTCCTGTACTGAGCTGCCC	11.84
hsa-miR-99a-5p	AACCCGTAGATCCGATCTTGTG	6.77
hsa-let-7 f-5p	GCCTGAGGTAGTAGATTGTATAG	6.61
hsa-miR-199a-3p>hsa-miR-199b-3p	ACAGTAGTCTGCACATTGGTTA	5.70
hsa-let-7 g-5p	TGAGGTAGTAGTTTGTACAGTT	3.38
hsa-let-7i-5p	TGAGGTAGTAGTTTGTGCTGTT	3.33
hsa-miR-100-5p	TACCCTGTAGAACCGAATTTGTG	2.52
hsa-miR-199b-5p	CCCAGTGTTTAGACTATCTGTTC	2.50
hsa-miR-26a-5p	GCTTCAAGTAATCCAGGATAGG	2.43
hsa-miR-191-5p	CAACGGAATCCCAAAAGCAGCTG	1.04
hsa-miR-143-3p	TGAGATGAAGCACTGTAGCTC	1.02
hsa-miR-10b-5p	TACCCTGTAGAACCGAATTTGTG	0.90
hsa-miR-144-3p	GCCTACAGTATAGATGATGTACT	0.36
hsa-miR-451a	AAACCGTTACCATTACTGAGTT	0.30
hsa-miR-199a-5p	CAGTGTTCAGACTACCTGTTC	0.15
hsa-miR-125a-5p	CTGAGACCCTTTAACCTGTGA	0.12

### Uptake of LF-EVs by cells

LF-EVs were incubated with DiR, DiIC18(7) [1,1ʹ-dioctadecytetramethyl indotricarbocyanine Iodide (cat. No. 125,964; PerkinElmer, Waltham, MA, USA)] for 20 min and excess dye from the labelled LF-EVs was removed by ultracentrifugation at 100,000 × *g* for 60 min at 4°C. The final pellets were resuspended in 0.9% N.S. for the internalization assay. Human umbilical vein endothelial cells (HUVECs) or ADSCs were co-cultured separately with labelled LF-EVs in the medium at 37°C. The uptake of LF-EVs was observed using a confocal laser scanning microscopy (Leica, Wetzlar, German) after 3 days.

### Cell proliferation assays

A Cell Counting Kit-8 assay (CCK8; cat. No.CK04; Dojindo, Kumamoto, Japan) was used to assess cell proliferation. HUVECs were trypsinized, seeded into 96-well tissue culture plates, and added with fetal bovine serum (FBS)-free medium. After overnight incubation, the cell culture medium was replaced and simultaneously added with 100 μg/mL of LF-EVs or 0.9% N.S. At 6, 24, 48, 72, 96, and 120 h, CCK8 solution (10 μL/well) was added to the cells. The absorbance was measured using a microplate reader. The optical density (OD) values represented the survival/proliferation of the cells.

### Cell scratch assays

The effects of LF-EVs on HUVECs migration were evaluated using cell scratch assays. HUVECs pretreated with LF-EVs (100 μg/mL) or an equal volume of 0.9% N.S. were plated in 12-well tissue culture plates and grown to form a confluent monolayer. The monolayer was scratched using a pipette tip in serum-free media supplemented with LF-EVs (100 μg/ml) or 0.9% N.S. The cells were incubated and photographed after 0, 6, 12, and 24 h. Wound areas were measured using ImageJ software (NIH, Bethesda, MD, USA).

### Tube formation assay

Capillary-network formation was monitored by performing tube formation assays. HUVECs pretreated with LF-EVs (100 μg/mL) or an equal volume of 0.9% N.S. were seeded onto 48-well plates precoated with Matrigel (cat. No.356234; BD Bioscience, San Jose, CA, USA). Tube formation ability was examined using phase-contrast microscopy at 2, 4, and 8 h. The total number of tube structures was measured per well and per high-power field using ImageJ software.

### Adipogenic differentiation assays

ADSCs were isolated from human subcutaneous adipose tissue and identified according to previously reported methods [19]. Then, assays for adipogenic differentiation potential were performed according to the manufacturer’s protocol (cat. No. HUXMD-90,031; Cyagen Biosciences, Inc.). Briefly, ADSCs at passage four were seeded in six-well tissue culture plates. When the cells reached 80% confluence, the medium was replaced with adipogenic differentiation medium A. After using medium A for 3 days, adipogenic maintenance medium B was replaced for 24 h. After using medium A and B alternately three times (about 12 days of culture), cells were stained with Oil Red O according to the following method. Cells were fixed with 4% paraformaldehyde for 20 min and then stained with Oil Red O for 15 min at room temperature. Induced fat cells contain orange-red oil droplets. The cells were washed with distilled water to remove excess dye and observed and photographed under a microscope. 2 ml isopropanol was then added to each well and left at room temperature for 1 hour. When the oil red O was dissolved in the isopropanol solution, the absorbance was detected at 500 nm using a spectrophotometer. Meanwhile, total proteins in the duplicate wells were extracted with a RIPA lysis buffer (cat. No. C1055; Applygen Technologies Inc.) and quantified using a bicinchoninic acid protein assay kit (cat. No. CW0014S; CW biotech, Beijing, China).

### Animal experiments

Forty-eight 6-week-old (bodyweight of 18–22 g), female BALB/c nude mice were purchased from the experimental animal centre of Peking University Health Science Center. All applicable institutional and/or national guidelines for the care and use of animals were followed in this experiment, which was granted ethics approval (No. LA2018329).

To observe the uptake of LF-EVs by cells *in vivo*, eight mice were used in this part experiment and two groups were set up. After intraperitoneal injection anaesthesia using 2% chloral hydrate at 0.0125 mL/g body weight, 0.3 mL of fat granules mixed with 40 μg of DiR-labelled LF-EVs in 20 μL 0.9% N.S or an equal volume of 0.9% N.S were injected subcutaneously into the back of the nude mice. Then, *in vivo* images were obtained using the IVIS Spectrum In Vivo Imaging System (PerkinElmer) at 3, 7, 14, and 28 days after fat transfer. On the 14th day, two mice in each group were sacrificed and the fat grafts on both sides of the mice were harvested and analysed as frozen sections. The red fluorescence in the cells of the local tissue was observed using confocal laser scanning microscopy (Leica).

To study the validity of LF-EVs-assisted fat transplantation, forty mice were used in this part and two groups were set up: The LF-EVs group and the control group. Fat granules (0.3 mL) mixed with 40 μg of LF-EVs in 20 μL 0.9% N.S or an equal volume of 0.9% N.S (20 μL) were injected subcutaneously into the backs of nude mice using a 1 mL syringe, on both sides of the spine of each mouse. At day 7 and day 14, each transplanted fat bolus in the LF-EVs group was injected with 40 μg of LF-EVs, while the control group was injected with the same volume of 0.9% N.S. After 7, 14, 28 and 56 days of fat transfer, five mice of each group were sacrificed and the fat grafts on both sides of the mice were harvested, weighed, and analysed(Table 2).

**Table 2.** Day-wise schedule of LF-EVs-assisted fat transplantation

Group	Day0	Day7	Day14	Day28	Day56
LF-EVs	Fat granules +LF-EVs(n = 20)	Sacrificed (n = 5); +LF-EVs(n = 15)	Sacrificed (n = 5); +LF-EVs(n = 10)	Sacrificed(n = 5)	Sacrificed (n = 5)
Control	Fat granules + N.S(n = 20)	Sacrificed (n = 5); +N.S (n = 15)	Sacrificed (n = 5); + N.S (n = 10)	Sacrificed(n = 5)	Sacrificed (n = 5)

### Histological analysis

Paraffin-embedded tissue sections were stained with haematoxylin and eosin (HE) to calculate the percentage area of vacuoles. Masson staining was used to indicate fibrotic areas and perilipin-1 (cat. No. 9349; Cell Signalling Technology, Danvers, MA, USA) immunohistochemistry was used to determine the structural integrity of adipocytes. All measurements were performed using ImageJ software.

## Quantitative real-time reverse transcription polymerase chain reaction (qRT-PCR)

The harvested fat grafts were snap-frozen in liquid nitrogen, and total RNA was extracted using Trizol® (cat. no. 15,596,026; Thermo Fisher Scientific, Inc.). Then, cDNA was synthesized using Evo M-MLV RT Kit with gDNA Clean for qPCR (cat. no. AG11705; Accurate Biotechnology Co. Ltd.), and PCR was performed using SYBR® Green Premix *Pro Taq* HS qPCR Kit (cat. no. AG11701; Accurate Biotechnology Co. Ltd.) according to the manufacturer’s instructions. The primers used for real-time PCR are shown in Table 3. The amount of each target gene was normalized to the expression of the housekeeping gene *GAPDH*.

**Table 3.** Primers for the real-time polymerase chain reaction of the fat grafts

Gene name	Primers
HGF	F:GTCCTGAAGGCTCAGACTTGGT
	R:CCAGCCGTAAATACTGCAAGTGG
FGF2	F:AAGCGGCTCTACTGCAAGAACG
	R:CCTTGATAGACACAACTCCTCTC
VEGFA	F:CTGCTGTAACGATGAAGCCCTG
	R:GCTGTAGGAAGCTCATCTCTCC
ANG	F:GAGCGAATGGAAGCCCTTACAG
	R:GGCAAGCCATTCTCACAGGCAA
PPARG	F:GTACTGTCGGTTTCAGAAGTGCC
	R:ATCTCCGCCAACAGCTTCTCCT
LPL	F:GCGTAGCAGGAAGTCTGACCAA
	R:AGCGTCATCAGGAGAAAGGCGA
CEBPB	F:CAACCTGGAGACGCAGCACAAG
	R:GCTTGAACAAGTTCCGCAGGGT
GAPDH	F:GAGAGTGTTTCCTCGTCCCG
	R:ACTGTGCCGTTGAATTTGCC

### Statistical analysis

Data are shown as means ± standard deviation (SD). Statistical analysis was performed using SPSS 16.0 software package (IBM Corp. Armonk, NY, USA). For comparison between two groups, independent sample t-tests were performed. P < 0.05 was considered statistically significant.

## Results

### Characterization of LF-EVs

**Figure 2.**
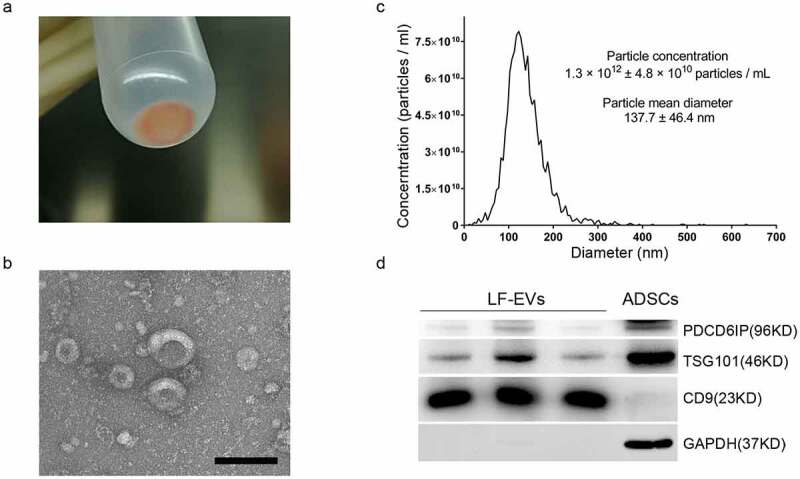
Characterization of LF-EVs. (a) The semitransparent gelatinous appearance of the obtained pellet (LF-EVs) after ultracentrifugation. (b) Transmission electron micrograph of the LF-EVs revealed cup-shaped structures with different sizes. Scale bar = 200 nm. (c) The size distribution and concentration of LF-EVs as determined by nanoparticle tracking analysis (NTA). (d) Identification of LF-EVs by the detection of TSG101, CD9, and GAPDH with 20 μg of proteins loaded per lane. Compared with ADSCs, CD9 is highly abundant in LF-EVs, with only a small amount of GAPDH; while GAPDH and TSG101 are highly abundant in ADSCs, with little CD9. LF-EVs, extracellular vesicles derived from lipoaspirate fluid; ADSCs, adipose tissue-derived stem cells

The appearance of the obtained pellet (LF-EVs) after ultracentrifugation was a semitransparent gelatinous precipitate in red-yellow colour (Figure 2a). Usually, 2–10 mg of LF-EVs could be extracted from 100 mL of liposuction solution. Under the transmission electron microscope, the LF-EVs were mostly cup-shaped or spherical with different sizes (Figure 2b). As shown in Figure 2c, the mean particle diameter of the LF-EVs was 137.7 ± 46.4 nm, the diameter of most particles (96.7%) was 122.1 nm, and the particle concentration was 1.3 × 10^12^ ± 4.8 × 10^10^ particles/mL. The characteristics of LF-EVs were further validated using western blotting to detect the EV markers PDCD6IP, TSG101 and CD9. The results showed high levels of CD9 in LF-EVs, with a small amount of cell marker GAPDH. Meanwhile, while GAPDH and TSG101 were highly expressed in ADSCs, their content of CD9 was very low (Figure 2d).

### Bioinformatic analysis of proteins enriched in LF-EVs

Proteins identified by both biological repetitions of the three LF-EVs samples and with at least two unique peptides were chosen manually to create the final protein list. Finally, 1185 nonredundant quantifiable co-expressed protein groups corresponding to 1242 gene symbols were identified.

**Figure 3.**
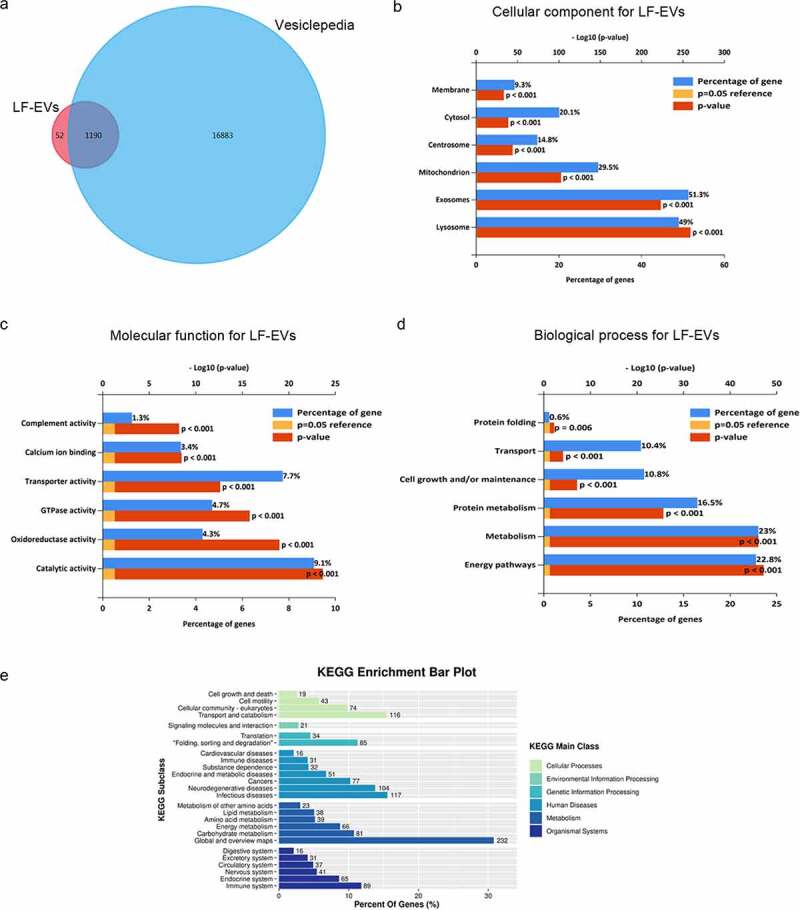
Bioinformatic analysis of proteins enriched in LF-EVs. (a) Venn diagram illustrating that of the 95.81% gene symbols in LF-EVs overlapped with entries in the Vesiclepedia database. b-d. GO analysis to group the identified proteins. For each GO term (‘Cellular compartment’ (b), ‘Molecular function’ (c), ‘Biological process’ (d)), six categories with the highest statistical significance of protein enrichment within the given category (calculated as −log10(p-value)) are presented on the graphs.(e) KEGG pathway enrichment bar plot. Different colours correspond to different main classes. The y-axis shows the KEGG_Subclasses. The data corresponding to the x-axis indicates the percentage of genes with this role in the pathway among the total identified genes; and the number of each line represents the number of identified genes enriched for each KEGG_Subclass. LF-EVs, extracellular vesicles derived from lipoaspirate fluid; GO, gene ontology; KEGG, Kyoto encyclopaedia of Genes and Genomes

Venn diagrams, including Vesiclepedia protein overlap (Figure 3a), and gene ontology (GO) analysis (Figure 3b-d), regarding the cellular compartment, molecular function, and biological processes were performed using the FunRich 3.1.3 software, with the FunRich database as a reference. A comparison with the contents of the Vesiclepedia database disclosed that 1190 (95.81%) gene symbols identified in the present study were also detected by other vesicle-related studies, supporting their vesicular origin. In addition, the proteins identified in the LF-EVs confirmed the presence of common exosome markers, CD9, CD63, CD81, and programmed cell death 6 interacting protein (PDCD6IP), as well as specific adipogenic molecules, e.g. adiponectin, C1Q and collagen domain containing (ADIPOQ), adipogenesis regulatory factor (ADIRF), fatty acid binding protein 4 (FABP4), fatty acid binding protein 5 (FABP5), lipoprotein lipase (LPL), and Perilipin 1 (PLIN1). The largest group of LF-EVs proteins was associated with exosomes (up to 51.3% of the identified proteins) or lysosomes (up to 49%, Figure 3b). This finding reflects the mechanism of LF-EVs biogenesis. Moreover, many of the identified proteins were associated with mitochondria (up to 29.5%), and approximately 14.8% of the proteins were related to centrosomes, reflecting the accumulation of their components in LF-EVs. Regarding the molecular functions (Figure 3c), the most abundant groups of proteins were related to catalytic activity, transporter activity, GTPase activity, and oxidoreductase activity. Annotations related to biological processes (Figure 3d) revealed enrichment for proteins involved in metabolism, energy pathways, protein metabolism, and cell growth and/or maintenance. Additionally, protein-enriched pathways were generated via Kyoto Encyclopaedia of Genes and Genomes(KEGG) analysis. A total of 754 proteins were identified via KEGG annotation and mapped to 84 pathways. As shown in Figure 3e, the identified proteins participated in six KEGG main classes, of which the most enriched was ‘global and overview maps’ in metabolism pathways. Other metabolism subclasses include ‘lipid metabolism’, ‘amino acid metabolism’, ‘carbohydrate metabolism’, and ‘energy metabolism’. In cellular processes, the enriched subclasses were ‘cell growth and death’, ‘cell motility’, ‘cellular community’, and ‘transport and catabolism’.

The main sources of LF-EVs could be components of fat tissues (including SVF cells, mature adipocytes, and blood circulation cells); therefore, we speculated that LF-EVs would have markers of fat cells, ADSCs, T lymphocytes, platelets, and vascular endothelial cells etc. To validate this speculation, after downloading the file of Human_cell_markers from CellMarker database [20] (http://bio-bigdata.hrbmu.edu.cn/CellMarker/download/Human_cell_markers.txt), we compared the corresponding 1242 gene symbols of total identified proteins with the gene symbols of normal cell markers in adipose tissue and blood, and found that the LF-EVs indeed contain certain markers present in normal adipose tissue derived mesenchymal stem cells, endothelial cells, endothelial precursor cells, platelets or megakaryocytes, fat cells (adipocytes), preadipocytes, dedifferentiation adipocytes, haematopoietic stem cells, circulating precursor or progenitor cells, dendritic cells, leukocytes, macrophages or monocytes, T cells, lymphoid cells other than T cells, fibroblasts, and red blood cells (erythrocytes) (Table 4).

**Table 4.** Comparison results between identified proteins with gene symbols of normal cell markers in adipose tissue and blood in CellMarker database

Cell Name	Count(C)		Gene Symbols of cell markers expressed in LF-EVs	Total number of cell markers in CellMarker database(TN)	Rich factor(C/TN)
Normal Adipose tissue derived mesenchymal stem cell	15		THY1,ITGB1,ANPEP,CD34,CD44,MME,MCAM,PDGFRB,NT5E,CD9,CD59,TFRC,CD14,ITGA5,ITGAV	61	0.245902
Endothelial cell or Endothelial precursor cell	19		MCAM,CD34,ANPEP,MRC1,CD81,ACE,BSG,CD151,CD36,COLEC12,FABP5,ITGB1,TYMP,STAB1,ITGB3,RFTN1,CD14,ITGA5,CSPG4,	92	0.206522
Platelet or megakaryocyte	15		ITGA2B,CD14,ITGB3,LAMP2,BSG,CD151,ITGB1,CD36,CD47,ITGAV,CD63,CD9,CD34,PF4,TFRC,	48	0.3125
Fat cell (adipocyte),Preadipocyte,or Dedifferentiation adipocyte	7		ADIPOQ,FABP4,SLC2A4,CD36,CD34,ITGB1,CD44	34	0.205882
Haematopoietic stem cell	4		CD34,ALDH1A1,CD44,THY1	39	0.102564
Circulating precursor or progenitor cell	3		CD34,CD14,MCAM	6	0.5
Dendritic cell	109		ACTN1,ANPEP,APOL1,CAMK2D,CD59,CPNE3,CYB5R3,DPP4,EHD4,ELOVL5,ENPP1,INF2,LMNA,NCALD,PPA1,ITGAM,CD14,ITGA5,PEA15,ANXA1,BST1,CD163,CD36,CES1,F13A1,MGST1,S100A8,S100A9,AGTRAP,AP2A1,ASAH1,CKB,EHD1,FLNA,FTH1,FTL,GIMAP1,GLUL,GNG2,GNS,HCK,HK1,HMOX1,ITGB1,LGALS3,LIMS1,LRP1,LYN,MAGED2,MARCKS,MSN,MYOF,NAP1L1,NDUFB3,PLEKHO2,PSAP,PYGL,RAB10,RAP1B,S100A11,S100A4,SDCBP,SERPINA1,TAGLN,TBXAS1,TKT,TMBIM1,TYMP,ALDH2,ATP2B4,DPYSL2,GNAQ,MGLL,MYH11,S100A10,SUCLA2,TXN,CST3,ABHD15,ADA,AHCY,ASPH,ATP2A3,CNP,CTSB,DHRS7,ERP29,FKBP2,FLNB,HSP90B1,IDH3A,LMAN1,NUCB2,P4HB,PARK7,PCYOX1,PDIA4,PFKP,PLP2,SCARB2,SERPINF1,SLC2A1,SLC3A2,SND1,SSR4,STT3A,TGFBI,TUBB6,TXNDC5	892	0.122197
Leukocyte including Neutrophil,Eosinophil,Basophil,etc.	7		CD63,ITGAM,CD14,CD44,CD81,MME,ANPEP	58	0.12069
Macrophage or monocyte	7		CD163,CD14,MRC1,CD36,ITGAM,MPO,BSG	89	0.078652
T cell	25		ITGB1,TFRC,CD14,SLC2A1,ITGAM,AHNAK,CAPN2,DSTN,FLNA,GLUL,LGALS1,RAP1B,RAP2B,TAGLN2,VCL,ACTN1,EEF1B2,RPS5,CPNE2,LMNA,METTL7A,SAMHD1,TTN,UCHL1,MCAM	215	0.116279
Lymphoid cell other than T cell	6		ANXA5,HPRT1,TAGLN2,MME,TFRC,ITGAM	62	0.096774
Fibroblast	1		THY1	4	0.25
Red blood cell (erythrocyte)	1		GYPA	5	0.2

### Profiling of miRNAs enriched in LF-EVs

**Figure 4.**
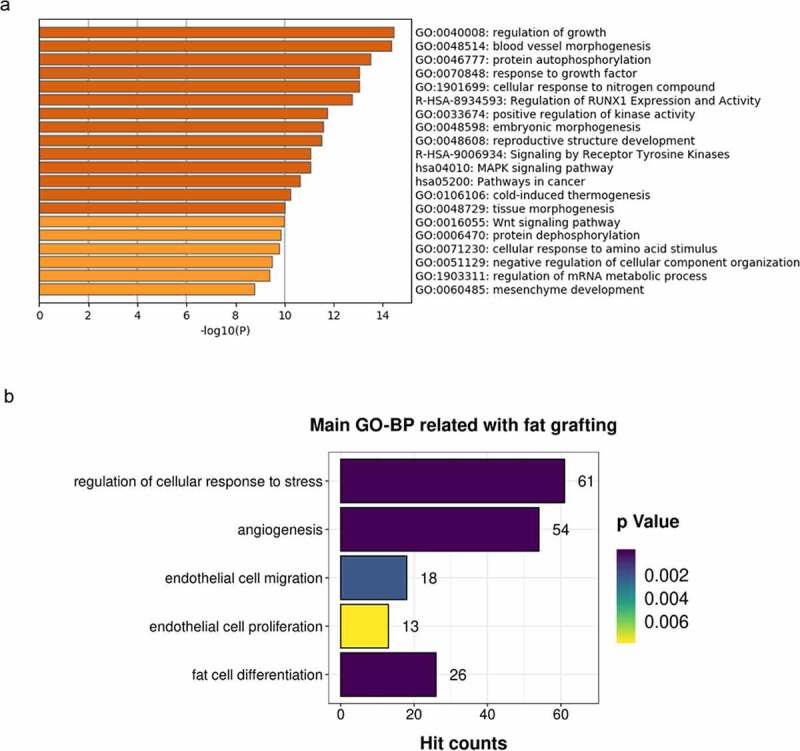
Enriched ontology clusters for target genes of the top 13 miRNAs (the same as those top 13 target microRNAs names listed in Table 1) enriched in LF-EVs. (a) Bar graph of the top 20 enriched terms in the Metascape Gene List Analysis Report, one row per cluster, coloured by p-values. (b) Enriched main GO Biological Processes terms related to fat grafting. LF-EVs, extracellular vesicles derived from lipoaspirate fluid; GO, gene ontology

The total number of known miRNAs identified in the small RNA-seq analysis was 737, and 89 new miRNAs were predicted from all samples. Among the 456 miRNAs that were present in all three samples, the total number of known miRNAs was 413, and there were 43 predicted novel miRNAs. We focused on the miRNAs present with the highest signal intensities (top 20) in all three samples and performed biological validation in ten human individuals using qRT-PCR. Finally, the top 13 miRNAs (hsa-let-7a-5p, hsa-let-7b-5p, hsa-let-7 c-5p, hsa-miR-125b-5p, hsa-miR-486-5p, hsa-miR-99a-5p, hsa-let-7 f-5p, hsa-miR-199a-3p>hsa-miR-199b-3p, hsa-let-7 g-5p, hsa-let-7i-5p, hsa-miR-100-5p, hsa-miR-199b-5p, and hsa-miR-26a-5p) were selected for bioinformatic analysis. Predicted target mRNAs for these top 13 miRNAs were identified using mirDIP 4.1 software [21]. For the 727 non-redundant target genes obtained with an integrated score greater than 0.8 in mirDIP, online cluster analysis was carried out using Metascape [22](http://metascape.org/). As shown in Figure 4a, the most significantly enriched top two clusters were regulation of growth (Hit count: 63) and blood vessel morphogenesis (Hit count: 63). Other enriched clusters related to fat grafting included regulation of cellular response to stress, angiogenesis, endothelial cell migration, endothelial cell proliferation, and fat cell differentiation (Figure 4b).

### LF-EVs promoted the proliferation and migration of endothelial cells

**Figure 5.**
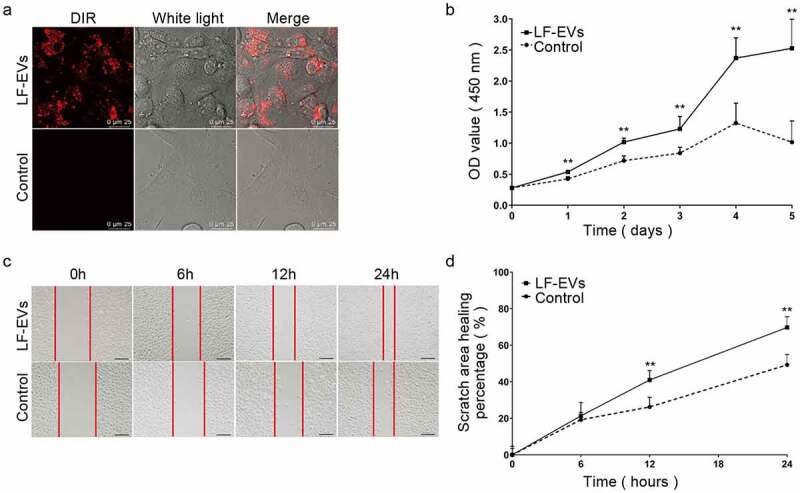
LF-EVs promoted the proliferation and migration of endothelial cells. (a) Representative fluorescence photomicrograph showing the internalization of DiR-labelled-LF-EVs (red) by HUVECs. Scale bar = 25 μm. (b) The proliferation of HUVECs measured using the CCK8 method; the optical density (OD) values from three individual experiments on day 1, 2, 3, 4, and 5 are provided. (c) The images of cell scratch test in HUVECs incubated with LF-EVs (100 μg/mL) or 0.9% normal saline (N.S.) for 6, 12, and 24 h. Bar = 200 μm. (d) The wound-healing percentage of HUVECs is presented as the migration area/original area. Note: (** *P* < 0.01). LF-EVs, extracellular vesicles derived from lipoaspirate fluid; DiR, DiIC18(7) (1,1ʹ-dioctadecytetramethyl indotricarbocyanine Iodide); HUVECs, human umbilical vein endothelial cells; CCK8, cell counting kit 8

Confocal microscopy analysis showed that the DiR-labelled LF-EVs were taken up and transferred to the cytoplasm of HUVECs, indicating that they could internalize the LF-EVs (Figure 5a).

LF-EVs treatment increased the proliferation of HUVECs, as determined using a CCK8 assay during 1–5 days of growth, compared with that of the control group (Figure 5b). The results of the scratch test showed that the migratory ability of HUVECs was significantly increased in the LF-EVs group at 12 and 24 h compared with that of the control group (Figure 5(c,d)).

### *LF-EVs induced angiogenesis and adipogenesis* in vitro

**Figure 6.**
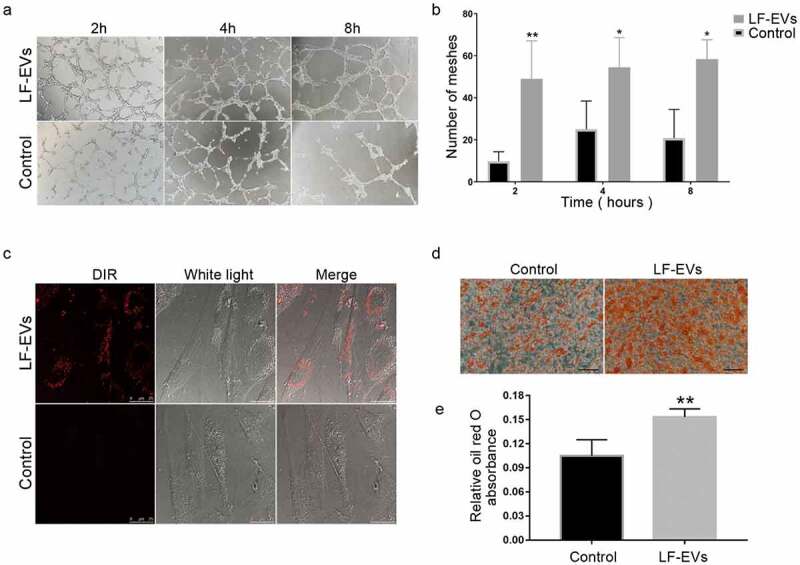
LF-EVs induced angiogenesis and adipogenesis *in vitro*. (a) Typical tube-like structures of HUVECs in matrigel in the two groups are shown. The formation of tubules could be observed after 2 hours and partially disintegrated after 8 hours. (b) The total number of tube structures per field of view from three experiments were analysed. (c) Representative fluorescence photomicrographs showing the internalization of DiR-labelled-LF-EVs (red) by the ADSCs. Scale bar = 25 μm. (d) Representative images of lipid droplets formed in human ADSCs induced with LF-EVs (100 μg/mL) or 0.9% normal saline (N.S.) are shown by staining with Oil Red O solution. Bar = 100 μm. (e) Semi-quantitative analysis of the relative Oil Red O absorbance per μg of protein in the two groups after adipogenic induction. Data in each bar chart represents the mean ± SD. Note: (* *P* < 0.05 and ** *P* < 0.01). LF-EVs, extracellular vesicles derived from lipoaspirate fluid; HUVECs, human umbilical vein endothelial cells; DiR, DiIC18(7) (1,1ʹ-dioctadecytetramethyl indotricarbocyanine Iodide); ADSCs, adipose tissue-derived stem cells

The angiogenic and adipogenic effects of LF-EVs were evaluated in HUVECs and ADSCs, respectively. After coculture with 100 μg/mL LF-EVs, the average numbers of tube structures within each field in the HUVECs were markedly higher than those in the control group (Figure 6(a,b)).

Similar to the HUVECs, confocal microscopy analysis showed that the DiR-labelled LF-EVs were taken up and transferred to the cytoplasm of ADSCs (Figure 6c). More lipid droplets were observed in the LF-EVs group than the control group when ADSCs were treated with 100 μg/mL LF-EVs during the process of inducing lipogenic differentiation. The absorbance of eluted Oil Red O per μg of protein was higher in the LF-EVs group than in the control group at 12 days (Figure 6(d,e)).

### *The uptake of DiR-labelled LF-EVs* in vivo

**Figure 7.**
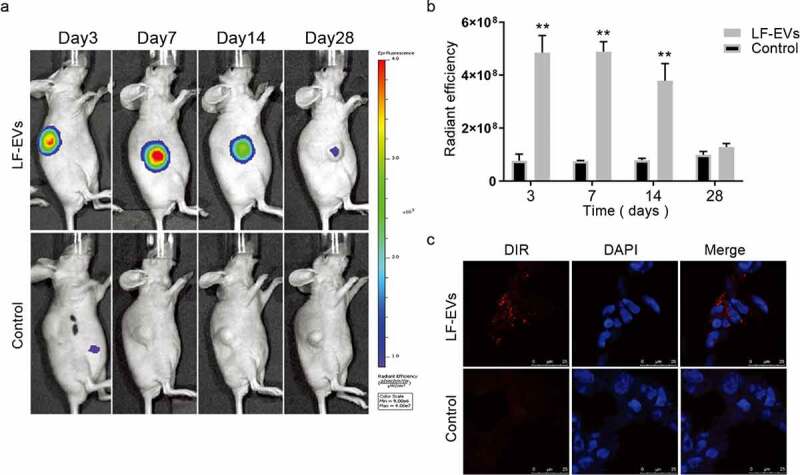
The uptake of DiR-labelled-LF-EVs *in vivo*. (a) Typical images of mice showing the uptake of DiR-labelled-LF-EVs in the fat tissue after local transplantation. (b) Total fluorescence intensity for DiR-labelled-LF-EVs was calculated in the region of the fat graft and results were expressed as total radiant efficiency [p/s/cm^2^/sr]/[μW/cm^2^]. (c) Representative fluorescence photomicrographs showing the internalization of DiR-labelled-LF-EVs (red) by the cells of the local fat graft (frozen section and nuclei were stained with DAPI) at the 14th day after transplantation. Scale bar = 25 μm. Note: (** *P* < 0.01). LF-EVs, extracellular vesicles derived from lipoaspirate fluid; DiR, DiIC18(7) (1,1ʹ-dioctadecytetramethyl indotricarbocyanine Iodide); DAPI, 4′,6-diamidino-2-phenylindole

After fat transplantation, the mice were imaged *in vivo* to observe the uptake of DiR-labelled-LF-EVs in the fat graft tissues. The uptake of LF-EVs in the local fat grafts was apparent on the 3rd, 7th, and 14 days after transplantation, which could be maintained to about 28 days after surgery (Figure 7(a,b)). On the 14th day, frozen sections showed that there was granular red fluorescence in the cells of the local fat graft (Figure 7c). The results suggested that LF-EVs could be retained locally and absorbed by cells over a short period.

### LF-EVs assisted fat transplantation

**Figure 8.**
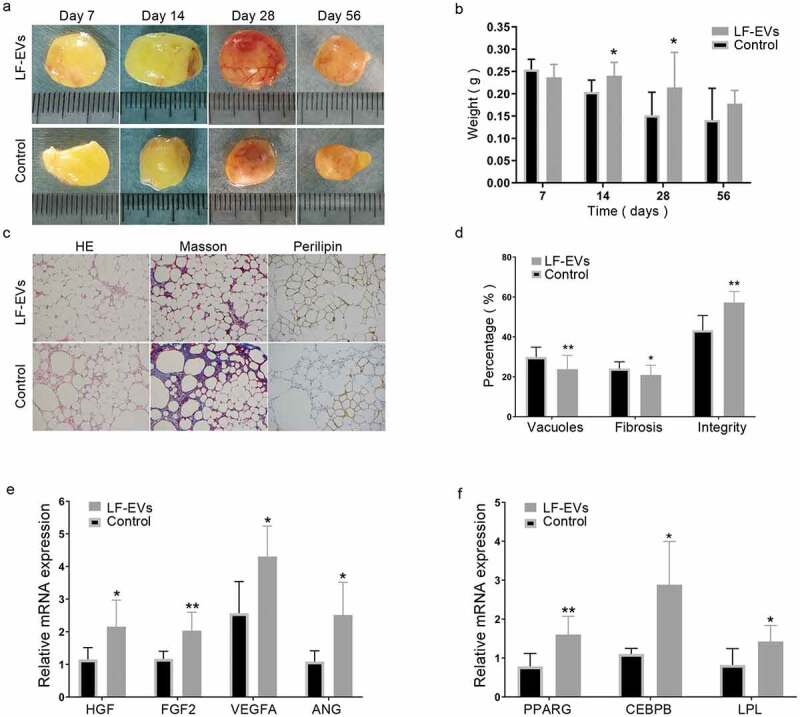
The effect of LF-EVs-assisted fat transplantation. (a) Gross appearance of fat grafts at different time points after the operation. Scale bar = 1 cm. (b) The weights of fat grafts at different time points. (c) Histological staining of the fat graft at 28 days after lipotransfer. Scale bar = 200 μm. (d) Histological analysis to evaluate the grafts at 28 days post-transplantation. The percentage areas of vacuoles, fibrosis, and cell structural integrity were calculated through HE, Masson, and perilipin-1 immunohistochemical staining, separately. (e) LF-EVs induced gene expression in transferred fat tissues at 14 days. Quantitative real-time PCR analysis of the mRNA expression of angiogenic factors (*Hgf, Fgf2, Vegfa, Ang*) and (f) adipogenic factors (*Pparg, Lpl, Cepbp*). Note: (* *P* < 0.05 and ** *P* < 0.01). LF-EVs, extracellular vesicles derived from lipoaspirate fluid; HE, haematoxylin and eosin

There was no significant difference between the two groups within 7 days, either in gross appearance or mean weight (Figure 8a). On days 14, 28, and 56 after surgery, the experimental group showed fewer oil cysts than the control group. In terms of the average weight, the LF-EVs group was significantly heavier than the control group on days 14 and 28, and although they were heavier on day 56, the difference was not significant (Figure 8b).

Histological assessment of the transferred fat

The histological characteristics of the transferred fat were examined at 28 days post-transfer. In the control group, the adipocytes had more vesiculation, local fibrosis, and inflammatory infiltration. In the LF-EVs group, the grafts showed better fat integrity, less fibrosis, and a larger area of perilipin-1 positive adipocytes (Figure 8(c,d)).

LF-EVs treatment upregulated the mRNA expression of genes related to angiogenesis and adipogenesis *in vivo*. Compared with those in the control group, the mRNA expression levels of angiogenic factors (hepatocyte growth factor (HGF), fibroblast growth factor 2 (FGF2), vascular endothelial growth factor A (VEGFA), and angiogenin (ANG)), and adipogenic factors (peroxisome proliferator activated receptor G (PPARG), LPL, and CCAAT enhancer binding protein beta (CEBPB)), as assessed by qRT-PCR, were significantly higher in the LF-EVs group compared with that in the control group in the transferred fat tissues at 14 days (Figure 8(e,f)).

## Discussion

The results of the present study confirmed, for the first time, that the adipose tissue fluid produced during liposuction operation contains abundant EVs, which can be obtained by ultracentrifugation. The LF-EVs can be taken up by cells *in vitro* and *in vivo*, and they have a strong ability to promote the proliferation, migration, and tube formation of vascular endothelial cells. In addition, the LF-EVs could promote the adipogenic differentiation of ADSCs *in vitro*. Multiple injections every 7 days promoted graft survival in a fat grafted mouse model. As far as we know, this is the first attempt to separate and prepare EVs from the adipose tissue fluid produced during tumescent liposuction surgery and their use to assist fat transplantation.

EVs are small membrane vesicles (30–200 nm in diameter) secreted by various cell types through the fusion of multi-vesicular bodies with the plasma membrane [8,23]. The secretion of EVs often increases during stress. The adipose tissue fluid produced during tumescent liposuction surgery contains the tumescence solution (containing saline, adrenaline, lidocaine, and sodium bicarbonate) injected into the adipose tissue before liposuction, the leaked fluid from adipose tissue and blood resulting from the injury caused by the liposuction surgical instruments and negative pressure injury. A special niche of ischaemia, hypoxia, and denervation forms during liposuction because of local anaesthesia, vasoconstriction, and short bursts of high pressure. As the negative pressure suction of fat increases, the mechanical trauma caused by the negative pressure and liposuction apparatus contributes to the microenvironment of acute ischaemia and hypoxia of subcutaneous tissue resulting from the sudden increase in extracellular space pressure. In the present study, we indeed extracted abundant LF-EVs from the fluid produced during the liposuction procedure.

The proteomic analysis indicated that the LF-EVs contain many homologs of common exosome markers [24]. In addition, the results shown in Table 3 indicated that the LF-EVs were probably derived from SVF cells, adipocytes, and blood circulation cells. In the KEGG analysis, the most enriched cluster was metabolic pathway (Figure 3d), which might reflect the metabolic state changes of the local cells to adapt to the change of the microenvironment and the metabolic regulation function of adipose tissue. In addition to their prominent role in fatty acid and energy metabolism, adipocytes and other cells in adipose tissue secrete a large number of different regulatory peptides (adipokines), as well as regulatory lipid species (lipokines). These factors can act on neighbouring cells (paracrine action) or on cells in other organs (endocrine action) to specifically regulate or modulate diverse processes, including lipid and glucose homoeostasis, energy balance, inflammation, and tissue repair [25,26]. In the present study, the identified proteins enriched in LF-EVs are involved in several KEGG main classes or subclasses, indicating the complex regulation process of adipose tissue to adapt the changed microenvironment. Apart from proteins, miRNAs are another component that are relatively stable and easy to transport via EVs. More than half of all human coding genes are under the control of miRNAs. In this study, we screened 13 miRNAs that were highly abundant in LF-EVs and are widely involved in the regulation of growth and blood vessel morphogenesis, cellular response to stress, angiogenesis, endothelial cell migration or proliferation, and fat cell differentiation. The results suggested that the LF-EVs might exert their regulatory functions by transferring specific miRNAs into cells.

Fat grafting technologies are popular in plastic and reconstructive surgery. In the past decades, the factors affecting the long-term survival of transplanted adipose tissue and the mechanisms to improve their interaction with surrounding tissues have been studied intensively [13,19]. We speculated that LF-EVs improved the survival rate of fat grafts by accelerating the reconstruction of the blood supply, promoting proliferation and differentiation of stem cells, reducing cell apoptosis, and regulating the immune response. Indeed, our research results demonstrated increased blood supply and adipogenic differentiation of ADSCs, both i*n vitro* and *in viv*o.

*In vitro*, LF-EVs promoted the proliferation, migration, and formation of tubules of HUVECs, as well as the adipogenic differentiation of ADSCs. In the mouse experiment, LF-EVs upregulated the expression of angiogenic factors (HGF, FGF2, VEGFA, and ANG), and adipogenic factors (PPARG, LPL, and CEBPB) in the fat grafts, suggesting that LF-EVs-assisted fat transplantation could promote angiogenesis and fat survival *in vivo*. These effects might be caused by factors or components derived from adipose tissue, which together create a suitable microenvironment for cell proliferation, migration, and differentiation, and induce the regeneration of blood vessels and fat tissue *in vivo*. Previous studies showed that adipose tissue can secrete a variety of angiogenic growth factors, such as angiopoietin-1 (ANGPT1), insulin-like growth factor 1 (IGF-1), vascular endothelial growth factor (VEGF), platelet-derived growth factor (PDGF), and HGF [27]. Herold et al. [25] demonstrated that keratinocyte growth factor (KGF), acidic fibroblast growth factor (aFGF), basic fibroblast growth factor (bFGF), and VEGF, as well as leptin and adiponectin, are contained in fat suspensions obtained by liposuction and in the supernatant. As expected, in studies using components from adipose sources to promote fat transplantation, researchers obtained similar results to those present here, although there is no standard method of obtaining a cell-free fat extract. Kelly et al [28]. found that bioactive adipose tissue extract can induce adipogenesis without additional stem cells or growth factors. Lopez et al [29]. showed that human adipose tissue extract (ATE) can induce angiogenesis and adipogenesis *in vitro*, and soft tissue formation *in vivo*.

Another possible way to realize the angiogenesis and adipogenesis functions of LF-EVs is the use of enriched miRNA. In terms of promoting blood vessel formation, EVs secreted from adipose-derived stem cells (ADSCs) contain multiple microRNAs including hsa-miR-125b-5p, hsa-miR-199a-3p, hsa-let-7 f-5p, hsa-let-7i-5p, etc. and promote the migration and invasion of HUVECs [30]. Plasmid overexpression of miRNA Let-7a in HUVECs induced robust tubule formation on an extracellular matrix gel [31]. EVs derived from ADSCs can transfer miR-125a to endothelial cells, which promoted angiogenesis by repressing *DLL4* expression [32]. Let-7b and miR-486 were also found in LF-EVs and have been demonstrated to promote angiogenesis [33]. In terms of adipogenic differentiation, miR-125a-3p and miR-483-5p promote adipogenesis by suppressing the RhoA/ROCK1/ERK1/2 pathway in multiple symmetric lipomatosis [34]. Moreover, miR-26a is a key regulator of human white and brite adipocyte differentiation and is upregulated in early adipogenesis [35]. In the present study, proteomic and miRNAs data analyses revealed enriched components of LF-EVs that regulated cell growth, angiogenesis, endothelial cell migration, endothelial cell proliferation, and fat cell differentiation, which might contribute to the mechanisms that promote fat graft survival.

This study had several limitations. Firstly, the source cells of the LF-EVs were not identified quantitatively, and the LF-EVs were a heterogeneous EVs population from a cocktail of cell sources. In order to reduce the steps of separation and preparation of LF-EVs and facilitate the clinical transformation, this experiment did not focus on how to obtain more purified LF-EVs. Secondly, it is not clear which proteins, lipids, or other signal molecules, such as miRNAs, play a major role in LF-EVs assisted fat transplantation. Thirdly, EVs provide a promising strategy for tissue regeneration; however, their short life span in tissues limits their practical use. Studies have shown that multiple carefully timed applications of EVs induced superior regeneration compared with that induced by a single dose of the same total concentration of EVs for diabetic and non-diabetic wounds, while the addition of hydrogel helped slow releasing EVs and provided a temporary extracellular matrix for cell infiltration and adhesion [36]. We have observed the effect of LF-EVs injection in fat transplantation only once, and the results showed that there was an increasing trend of the average graft weight in the experimental group, without statistical significance. According to the *in vivo* uptake experiment, the uptake peak period was about 7 days after transfer, and the effect of LF-EVs was markedly enhanced by supplementary injection every 7 days. Whether the combined transplantation of LF-EVs and hydrogel could enhance its biological effects remains to be determined. A further in-depth study of LF-EVs will be helpful for clinical translational application.

## Conclusions

The results of the present study showed that LF-EVs produced during tumescent liposuction could be considered as a new, easily accessible, and abundant source of EVs with potential value in adipose tissue regeneration or other regenerative therapies.

## Data Availability

The datasets used and/or analyzed during the present study are available from the corresponding author on reasonable request.
